# COVID-19 Infection Induces Rhabdomyolysis and Acute Kidney Injury

**DOI:** 10.7759/cureus.91153

**Published:** 2025-08-28

**Authors:** Mohamad Rababah, Amer Rababah, Baha Alsmadi

**Affiliations:** 1 Nephrology, King Abdullah University Hospital, Irbid, JOR; 2 General Practice, Jabal Al-Zaitoon Hospital, Zarqa, JOR; 3 Gastroenterology, James Cook University Hospital, Middlesbrough, GBR

**Keywords:** acute renal injury, coronavirus 2019 (covid-19), corona virus disease 2019 (covid-19), rhabdomyolysis, rhabdomyolysis causing acute kidney injury

## Abstract

Coronavirus disease 2019 (COVID-19) is an infectious illness caused by the SARS-CoV-2 virus. COVID-19 mainly affects the respiratory tract, and it can also lead to rhabdomyolysis and acute kidney injury as extrapulmonary complications. Rhabdomyolysis syndrome is caused by muscle necrosis and intracellular contents into the circulation. Rhabdomyolysis is frequently associated with trauma or crush injuries, and less frequently with infections. We report two cases of COVID-19-induced rhabdomyolysis at King Abdullah University Hospital in Jordan. Both patients presented at the emergency department with markedly elevated creatine phosphokinase levels, accompanied by breathlessness, cough, myalgias, and fatigue. They were admitted and treated with aggressive intravenous fluids. One patient progressed to respiratory failure, required mechanical ventilation, and died. The other responded well to treatment and made a full recovery. Medical professionals need to be aware that COVID-19 can lead to rhabdomyolysis and acute kidney injury, and in these situations, the patient needs to be appropriately treated and provided with fluids.

## Introduction

In December 2019, an outbreak of a novel coronavirus emerged in Wuhan, China, later identified as Severe Acute Respiratory Syndrome Coronavirus-2 (SARS-CoV-2) [1]. By March 11, 2020, the World Health Organization (WHO) officially classified the outbreak as a pandemic due to its rapid global dissemination [1]. Although COVID-19 frequently manifests as pulmonary symptoms, medical professionals are increasingly observing systemic consequences, including problems in the heart, brain, gastrointestinal tract, hematologic system, and kidneys [2,3].

Rhabdomyolysis is a clinical syndrome characterized by the destruction of skeletal muscle and the subsequent release of intracellular contents into the bloodstream [4]. It commonly presents with elevated creatine kinase levels, muscle pain, and, in some cases, myoglobinuria [4]. Although a universally accepted diagnostic threshold is lacking, rhabdomyolysis is often defined as a serum CK level exceeding 1,000 U/L, which is approximately five times the upper limit of normal [4].

Among viral etiologies, influenza A and B are the most frequently associated with rhabdomyolysis, followed by human immunodeficiency virus (HIV) and enteroviruses [5]. Rarely, coronaviruses, including SARS-CoV-2, have been implicated in its development [5,6]. We reported two cases of COVID-19-associated rhabdomyolysis, one of which required intensive care unit admission due to severe rhabdomyolysis complicated by acute renal failure.

## Case presentation

Case 1

A 58-year-old man with no previous medical disease presented to the emergency department with myalgia, nausea, vomiting, dry cough, breathlessness, and confusion.

On initial evaluation in the emergency department, the patient was diaphoretic, anxious, and tachypneic with a skin rash on both arms and legs. The other physical examination was unremarkable. O₂ saturation on room air was 84%; blood pressure, 93/61 mmHg; pulse, 105 bpm; temperature, 37.1 °C; and arterial blood gases, pH 7.46, PaCO₂ 25 mmHg, HCO₃ 17 mmol/L.

**Figure 1:**
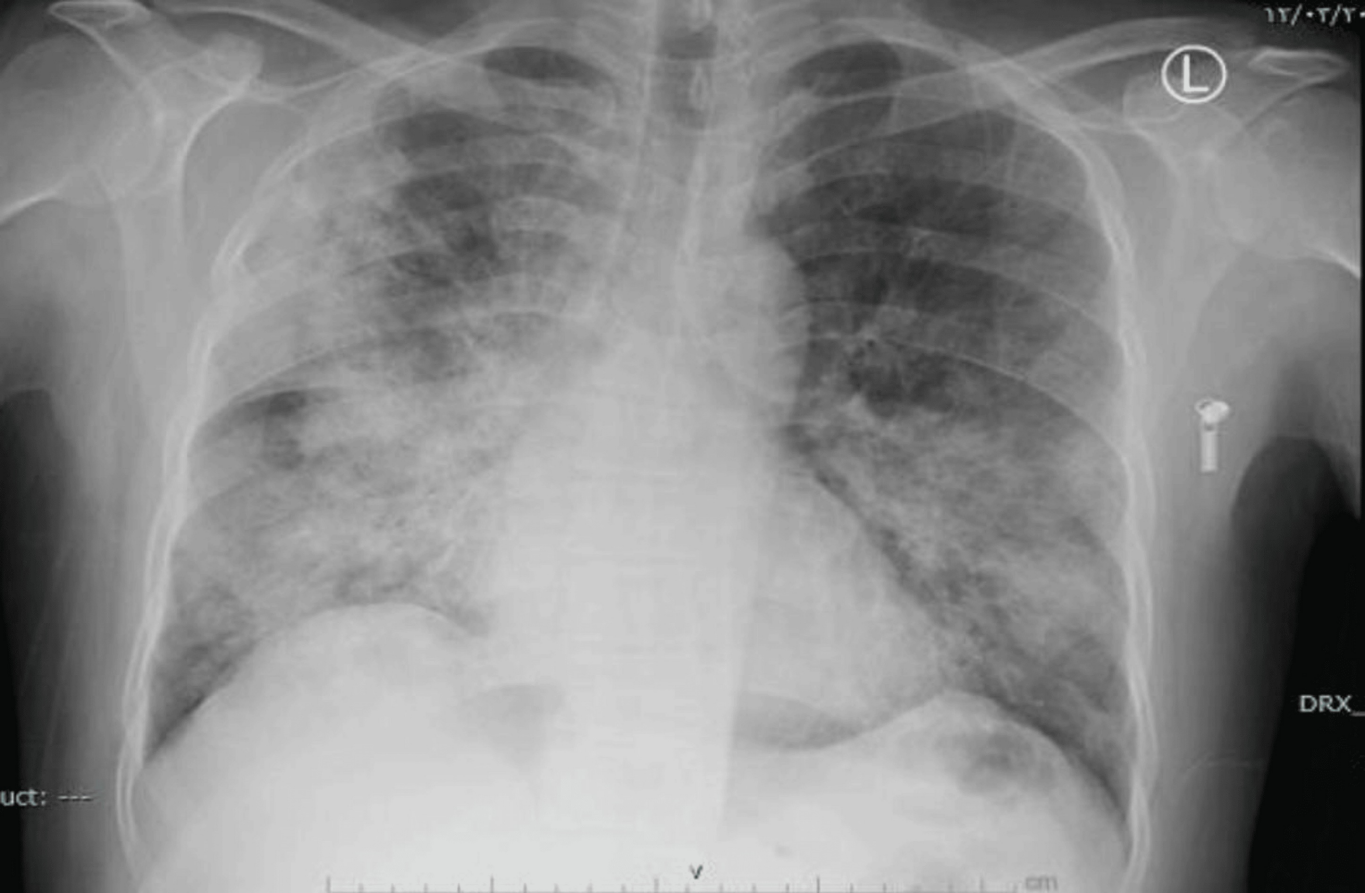
Chest X-ray on admission (Case 1)

**Figure 2:**
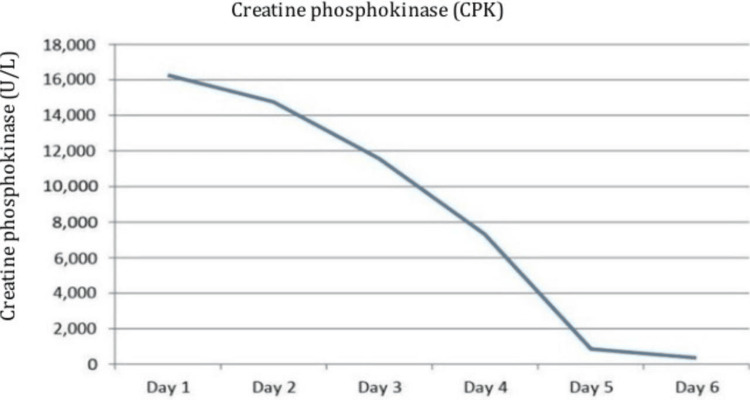
Creatine phosphokinase levels corresponding to the day of hospitalization (Case 1)

Initial labs revealed impaired kidney function with a serum creatinine of 154 micromoles/L, sodium 136 mEq/L, potassium 4.18 mEq/L, and a high level of serum creatine phosphokinase 16268 U/L. The polymerase chain reaction (PCR) was positive for COVID-19 infection. Other lab tests, Hb 14.6 g/dL, WBC's 8.4 × 10⁹/L, platelets 236 × 10⁹/L, C-reactive protein 222.5 mg/L, alanine transaminase (ALT) 112 U/L and aspartate aminotransferase (AST) 420 U/L. Laboratory investigations and their corresponding reference ranges are presented in Table 1. Chest X-ray performed on admission showed bilateral hazy infiltrates, as shown in Figure 1. On admission, the patient continued to receive aggressive intravenous fluid resuscitation and empiric antibiotics, and his creatine phosphokinase levels continued to normalize, as shown in Figure 2. On Day 8, the patient had fully recovered and was discharged.

**Table 1:** Laboratory investigations and reference ranges (Case 1)

Parameter	Result	Reference Range
Serum creatinine	154 µmol/L	60–110 µmol/L
Sodium	136 mEq/L	135–145 mEq/L
Potassium	4.18 mEq/L	3.5–5.0 mEq/L
Creatine phosphokinase	16268 U/L	20–200 U/L
PCR for COVID-19	Positive	-
Hemoglobin	14.6 g/dL	13.5–17.5 g/dL (male), 12.0–15.5 g/dL (female)
White blood cells	8.4 × 10⁹/L	4.0–11.0 × 10⁹/L
Platelets	236 × 10⁹/L	150–450 × 10⁹/L
C-reactive protein	222.5 mg/L	<5 mg/L
Alanine transaminase	112 U/L	7–56 U/L
Aspartate aminotransferase	420 U/L	10–40 U/L

Case 2

A 39-year-old man who was vaccinated with two doses of the Sinopharm vaccine and has a previous medical condition of diabetes mellitus type 2, presented to the emergency department with breathlessness, fever, rigors, myalgia, and a productive cough for seven days. The patient had contact with COVID-19 patients two weeks ago.

On initial evaluation in the emergency department, the patient was hypoxic O2 sat of 83% on room air, normotensive (106/63 mmHg), sinus rhythm (90 bpm), febrile (38.1 °C), and morbidly obese (BMI 42). His physical examination was unremarkable.

**Figure 3:**
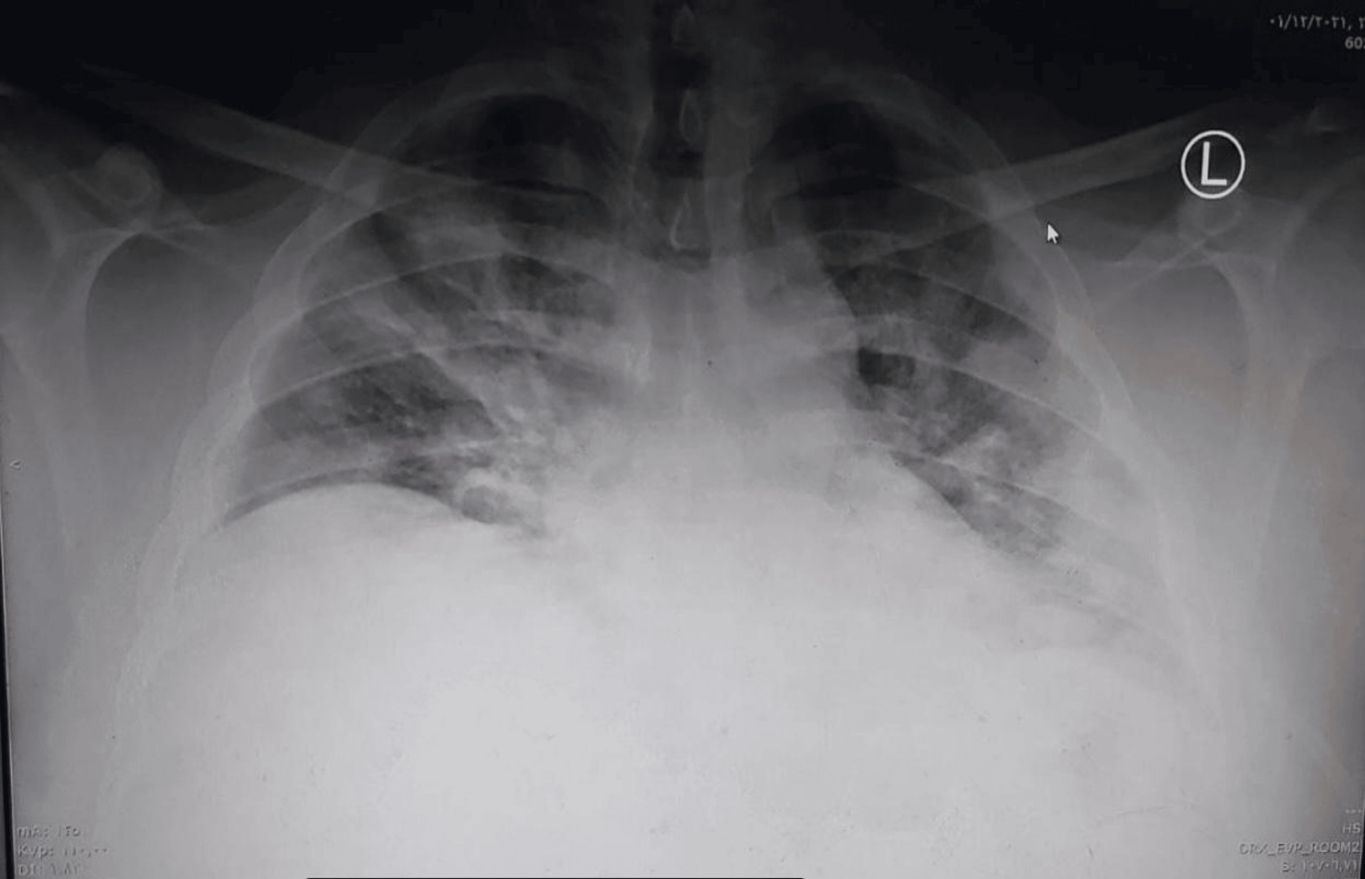
Chest X-ray on admission (Case 2)

Initial labs revealed impaired kidney function with a serum creatinine of 124 micromoles/L, urea 5.1 mmol/l, sodium 123 mEq/L, potassium 3.61 mEq/L, and a high level of serum creatine phosphokinase 7575 U/L. A nasopharyngeal swab for SARS-CoV-2 infection by polymerase chain reaction (PCR) was positive. Other lab tests showed Hb 13.4 g/dl, HCT 37.6, WBCs 7.4 × 10⁹/L, platelets 158 × 10⁹/L, lymphocytes 9%, neutrophils 89%, ferritin 2849 ng/ml, C-reactive protein 311.61 mg/L, lactate dehydrogenase (LDH) 1035 U/L, aspartate aminotransferase (AST) 118.7 U/L, and alanine transaminase (ALT) 43.6 U/L. Laboratory investigations and their corresponding reference ranges are presented in Table 2. Chest X-ray showed bilateral diffuse pulmonary infiltrates, as shown in Figure 3.

**Table 2:** Laboratory investigations and reference ranges (Case 2)

Parameter	Result	Reference Range
Serum creatinine	124 µmol/L	60–110 µmol/L
Urea	5.1 mmol/L	2.5–7.8 mmol/L
Sodium	123 mEq/L	135–145 mEq/L
Potassium	3.61 mEq/L	3.5–5.0 mEq/L
Creatine phosphokinase	7575 U/L	20–200 U/L
PCR for COVID-19	Positive	-
Hemoglobin	13.4 g/dL	13.5–17.5 g/dL (male), 12.0–15.5 g/dL (female)
Hematocrit	37.6%	38–50% (male), 34–44% (female)
White blood cells	7.4 × 10⁹/L	4.0–11.0 × 10⁹/L
Platelets	158 × 10⁹/L	150–450 × 10⁹/L
Lymphocytes	9%	20–40%
Neutrophils	89%	40–70%
Ferritin	2849 ng/mL	30–400 ng/mL
C-reactive protein	311.61 mg/L	<5 mg/L
Lactate dehydrogenase	1035 U/L	140–180 U/L
Aspartate aminotransferase	118.7 U/L	10–40 U/L
Alanine transaminase	43.6 U/L	7–56 U/L

**Figure 4:**
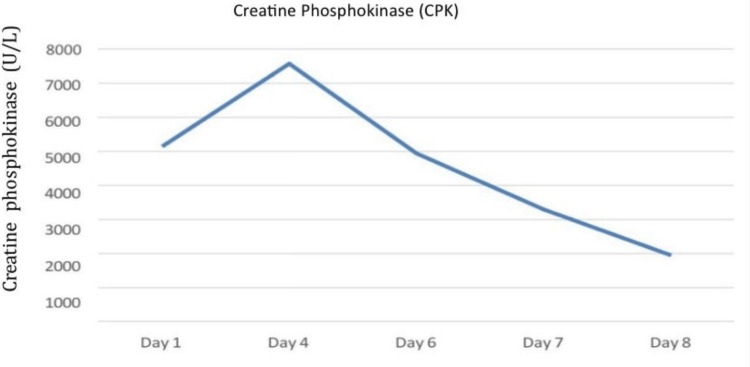
Creatine phosphokinase levels corresponding to the day of hospitalization (Case 2)

**Figure 5:**
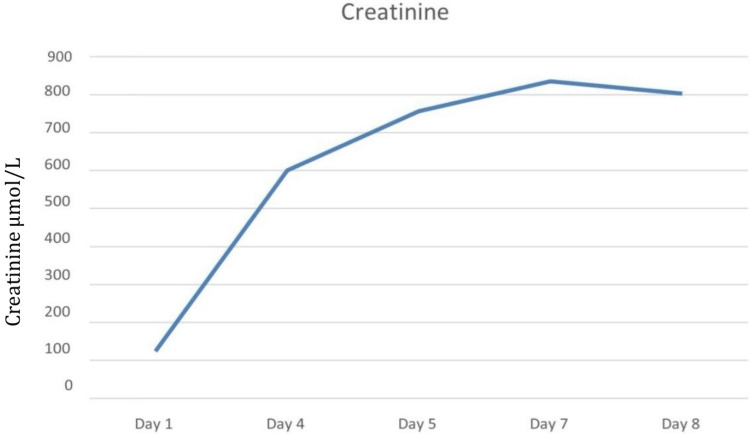
Creatinine levels corresponding to the day of hospitalization (Case 2)

On admission, the patient received aggressive IV fluids, normal saline 0.9%, sodium bicarbonate, and antibiotics. His creatinine phosphokinase and creatinine levels are shown in Figures 4-5.

The patient was transferred from the emergency department to the ICU and was initiated on mechanical ventilation on the second day of hospitalization. During the ICU stay, he developed a progressive rise in serum creatinine levels, reduced urine output, and clinical signs of volume overload. Consequently, renal replacement therapy was initiated, and the patient underwent a total of six sessions of hemodialysis. Despite intensive supportive measures, he passed away on the ninth day of hospitalization due to complications related to COVID-19 infection.

## Discussion

Renal involvement in the form of acute kidney injury is a well-recognized outcome of coronavirus disease 2019 (COVID-19) infection [7]. We report two patients with confirmed COVID-19 infection who developed renal impairment secondary to the infection. In one observational study of 5,449 hospitalized patients, 36.6% developed acute kidney injury and 14.3% required dialysis [8]. In our report, the patient in Case 2, who developed rhabdomyolysis and acute kidney injury, became anuric with signs of volume overload, required and underwent sessions of hemodialysis, and subsequently died due to complications of COVID-19. Crucially, the mortality rate among patients with acute kidney injury has been reported as 35%, compared with 16.3% in those without acute kidney injury [8].

The molecular mechanisms proposed for viral-induced rhabdomyolysis include: (1) direct viral invasion of muscle fibers, (2) cytokine-mediated muscle damage, and (3) circulating viral toxins causing myocyte membrane injury [9,10]. Myalgia, tea-colored urine, and muscle weakness are characteristic features of COVID-19-associated rhabdomyolysis [11]. Both of our patients presented with myalgia and had markedly elevated creatine phosphokinase levels alongside raised serum creatinine, indicating severe muscle injury with a high risk of rhabdomyolysis-associated acute kidney injury.

Patients with COVID-19 and rhabdomyolysis have been shown to have higher rates of ICU admission, mechanical ventilation, and mortality [12]. In Case 2, progressive respiratory failure necessitated ICU admission and invasive mechanical ventilation. In a large Chinese cohort of 1,099 COVID-19 patients, 14.9% reported myalgia and 0.2% developed rhabdomyolysis [13,14]. Our two cases provide further evidence linking COVID-19 to both renal impairment and rhabdomyolysis, highlighting the need for early recognition, aggressive fluid resuscitation, nephrotoxin avoidance, and close monitoring of renal function to prevent progression to dialysis and reduce mortality.

## Conclusions

Among the two patients in whom rhabdomyolysis occurred following clinical manifestations of COVID-19 infection (including pneumonia on radiography), all patients had significant muscle injury noted at the time of admission. Other possible causes of rhabdomyolysis have been ruled out; They denied engaging in rigorous activity or excessive exercise in the days before admission, and a physical examination revealed no signs of injuries. No exposure to myotoxic agents was identified in either patient upon evaluation. Subclinical hereditary myopathy has not been excluded from any of them. One patient had no prior medical conditions and made a full recovery, being discharged on Day 8. The other patient, who had type 2 diabetes mellitus, died due to complications of COVID-19.

Patients with COVID-19 infection may initially present with rhabdomyolysis and acute kidney injury. We suggest including creatine phosphokinase levels among the initial investigations when evaluating suspected COVID-19 cases or assessing acute kidney injury in patients with COVID-19. Early treatment with aggressive fluid replacement, monitoring electrolytes and urine output, and correcting acid-base imbalance can improve and prevent kidney injury.
